# Pore-scale characteristics of multiphase flow in heterogeneous porous media using the lattice Boltzmann method

**DOI:** 10.1038/s41598-019-39741-x

**Published:** 2019-03-04

**Authors:** Sahar Bakhshian, Seyyed A. Hosseini, Nima Shokri

**Affiliations:** 1 Bureau of Economic Geology, Jackson School of Geosciences, The University of Texas at Austin, Austin, TX 78758 USA; 2 School of Chemical Engineering and Analytical Science, The University of Manchester, Manchester, UK

## Abstract

This study provides a pore-scale investigation of two-phase flow dynamics during primary drainage in a realistic heterogeneous rock sample. Using the lattice Boltzmann (LB) method, a series of three-dimensional (3D) immiscible displacement simulations are conducted and three typical flow patterns are identified and mapped on the capillary number (*Ca*)-viscosity ratio(*M*) phase diagram. We then investigate the effect of the viscosity ratio and capillary number on fluid saturation patterns and displacement stability in Tuscaloosa sandstone, which is taken from the Cranfield site. The dependence of the evolution of saturation, location of the displacement front, 3D displacement patterns and length of the center of mass of the invading fluid on the viscosity ratio and capillary number have been delineated. To gain a quantitative insight into the characteristics of the invasion morphology in 3D porous media, the fractal dimension *D*_*f*_ of the non-wetting phase displacement patterns during drainage has been computed for various viscosity ratios and capillary numbers. The logarithmic dependence of *D*_*f*_ on invading phase saturation appears to be the same for various capillary numbers and viscosity ratios and follows a universal relation.

## Introduction

The present results extend the fundamental understanding of two-phase immiscible displacement in a realistic rock sample, such as Tuscaloosa sandstone, and offer new insights regarding the behavior of a non-wetting phase, such as CO_2_, as well as storage efficiency in a sequestration site.

Sequestration of carbon dioxide (CO_2_) in geological formations is considered to be as one of the most promising and most feasible solutions to mitigating carbon emissions and, hence, global warming. Oil and gas reservoirs, unmineable coal beds and deep saline aquifers are possible geological media for the sequestration of CO_2_^1–9^. Due to the wide distribution and large potential storage capacity of deep saline aquifers, they are one of the most ideal candidates for CO_2_ storage^10,11^. Injection of supercritical CO_2_ into saline aquifers, displaces the resident brine and leads to fingering and complex displacement patterns, which is highly dependent on the strength of the viscous and capillary forces and heterogeneity of the geological medium^12–16^. Pore-scale fluid displacement patterns and invasion dynamics strongly affect the macroscopic response and behavior of the fluid front. Development of the pore-scale models for multiphase displacement is crucial for enhancing the prediction of the field-scale simulations^17–19^. Therefore, having a deep understanding of pore-scale immiscible displacement phenomena in porous media is of critical importance.

The fluid displacement processes in porous media are influenced by many parameters, such as viscosity and density ratio of fluids, wetting properties, interfacial tension, the flow rate of the invading fluid and the heterogeneity of the medium^18–26^. Lenormand *et al*.^19^ have studied the effect of viscous and capillary forces on drainage displacement processes and have found that the capillary number (*Ca*), which is the ratio of the viscous forces to capillary forces and the viscosity ratio (*M*) between the advancing non-wetting and displaced wetting fluids are the most significant parameters affecting the displacement pattern of two-phase flow. After conducting a large number of displacement experiments in micromodels, they proposed a log *M*-log *Ca* phase diagram to present different immiscible displacement patterns including stable displacement, capillary fingering and viscous fingering. Numerous experimental studies have been devoted to investigate the immiscible multiphase fluid flow and identify the macroscopic properties of flow through porous media^27–29^. The complex experimental approaches, however, are not effective in terms of time and cost and, quantifying some properties such as capillary and viscous pressure drops is not trivial^30^. On the other hand, a numerical approach for pore-scale processes is a powerful tool to predict the most appropriate macroscopic model and constitutive relationship for describing the behavior of any flow regime.

The conventional numerical methods for multiphase flow simulations include level set^31^, the volume of fluid^32^, phase-field^33^ and smoothed particle hydrodynamics (SPH) models^34^. The evolution of fluid-fluid interfacial dynamics in these methods is difficult to track and information at the small scale is mainly missing. To incorporate essential physics at the microscopic level, lattice Boltzmann modeling has emerged as a promising technique that is capable of simulating multiphase fluid flow in complex porous systems. In the LB model, the tracking or reconstruction of phase interfaces is avoided, whereas the interface can be maintained automatically through the kinetic approach and by considering intermolecular interactions^35,36^. There have been several standard LB models for the simulation of multiphase fluid flow. These models can be categorized as the colour-fluid model^37,38^, Shan and Chen’s potential model^39,40^, the free energy model^41^, the phase field-based model^42^ and the mean-field theory model^43^. The LB model that has been used in this study was initially proposed by Tölke *et al*.^44,45^. This model is an extension of the Colour-Gradient model introduced by Gunstensen *et al*.^38^ with improved numerical stability that can handle multiphase flow simulations with a low capillary number and a high viscosity ratio. While many studies in the literature have focused on multi-phase flow in a simple and homogeneous 2D porous media^13,46^, observing pore-scale fluid flow in a realistic three-dimensional (3D) porous model has received less attention^47–49^. Hence, our study aims at the dynamics of immiscible fluid displacement in a 3D natural porous sample.

In this paper, we present direct immiscible displacement simulations in three-dimensional micro-CT images of Tuscaloosa sandstone (presented in Supplementary Information Fig. S1), using a two-phase lattice Boltzmann method. The porous sample is initially saturated with a wetting phase, which is displaced by a non-wetting phase. The LB model is applied to efficient simulation of two-phase flow at a broad range of viscosity ratios and capillary numbers in a 3D natural rock. Different displacement patterns including, capillary fingering, viscous fingering and stable displacement have been identified and mapped on the phase diagram (*Ca*-*M* diagram) for immiscible displacement. Furthermore, We investigate the effect of the viscosity ratio and capillary number on the displacement characteristics of two-phase flow and quantify the saturation profile, front location and length of the center of mass for the non-wetting phase. In order to quantify the displacement patterns and the frontal shape, the fractal dimension of the non-wetting phase front and its dependency on saturation have been investigated. Our study provides information regarding the effect of three-dimensionality and pore heterogeneity on two-phase immiscible displacement in natural rocks and offers new insights on how to manage the reservoir conditions to gain the highest sweep efficiency during CO_2_ storage.

## Results and Discussion

### Two-phase displacement in a realistic rock model

We study two-phase flow and the drainage process of a non-wetting fluid into a three-dimensional (3D) heterogeneous porous medium. The 3D porous model used for the simulations is extracted from micro-CT images of a cylindrical core sample of Tuscaloosa sandstone taken by X-ray microtomography (XMT) with the resolution of 6.17 *μm* (Fig. S1). The image data exported from XMT are 16-bit grayscale image stacks, and the byte value of each pixel corresponds to the attenuation or density of the core sample at that point. The lower densities represent the pore space, whereas the higher values correspond to the solid matrix. Prior to applying the LB method to the 3D rock model, the image stacks are required to be processed by a segmentation process to precisely distinguish pores from minerals in the images. The segmentation is done using ImageJ. The threshold for the segmentation process is selected, as the binary image stack obtained has the same porosity as the original core samples (which is 26%). Consequently, the 3D binarized representation can be applied to the LB model for immiscible two-phase fluid flow simulation. The size of the computational domain is 200 × 200 × 200 *lu*^3^ (lu = lattice unit), as shown in Fig. S1. We did some preliminary simulations to define this representative elementary volume (REV) of the sample. In other words, we found the minimum image size such that its properties (including permeability and porosity) would not change significantly if larger volumes were used. The pore-size distribution of the sample has been shown in Fig. S2. The distribution confirms that there is a high degree of pore size variation within the rock sample. The distribution is statistically analyzed and its mean, standard deviation, skewness and kurtosis have been reported in Table S1 (Supplementary Information). The large value of standard deviation shows how much individual pore size within the sample differs from its average size. Skewness and kurtosis data were used to analyze the degree of dispersion in pore sizes. It is noticed that the pore size distribution is asymmetrical as it is skewed to the right (Fig. S2). The large value of kurtosis further underlines the high standard deviation observed in the pore-size distribution. We have also shown the distribution of porosity along the sample length (Fig. S3 in Supplementary Information). The variation of porosity along the sample represents its spatial heterogeneity.

Two buffer layers with a size of 10 *lu* are placed on the left and right boundaries. The left buffer layer is saturated with the non-wetting phase, whereas the right buffer layer is initially empty. The porous medium between these two layers is initially saturated with the wetting fluid. The non-wetting fluid is drained continuously with a constant flow rate from the left side of the packing, and a constant pressure is applied at the outlet on the right side. The other boundaries are assumed to be solid walls by imposing no-slip boundary condition through the bounce-back rule. In the following figures, the solid skeleton is represented with a gray color, the wetting phase is marked in red and the non-wetting phase is indicated in blue. In order to gain a better understanding of the characteristics of two-phase displacement in porous media, a series of LB simulations with different viscosity ratio (M = *μ*_*nw*_/*μ*_*w*_, where *μ*_*nw*_ and *μ*_*w*_ are the dynamic viscosity of the non-wetting and wetting fluids, respectively) and capillary number (*Ca*) have been carried out. The capillary number is defined as $Ca=\frac{{u}_{nw}{\mu }_{nw}}{\sigma }$, where *u*_*nw*_ is the average velocity of the non-wetting fluid^12,50^. In our numerical experiments, the densities of both the wetting and non-wetting fluids are considered to be unity. Since the displacement velocity in the simulations is sufficiently slow and the Mach number Ma is much less than one, the effect of compressibility on the flow patterns can be safely neglected^51^.

To improve the computational efficiency of the LB implementation, the model has been applied to a parallel scheme written in C++ using the Message Passing Interface (MPI). The simulations were carried out using 2 nodes of the HPC system at the Texas Advanced Computing Center at the University of Texas at Austin, with each node having 24 cores.

### Characterization of immiscible displacement patterns

A series of drainage simulations are performed in the rock sample considering viscosity ratios ranging from *M* = 0.067 to *M* = 100, and capillary numbers ranging from *Ca* = 5.8e-3 to *Ca* = 2.78. The rock model is assumed to be completely hydrophilic. A combination of various injection rate, pressure gradient, fluid viscosity and surface tension have been applied to perform two-phase immiscible displacement simulations under a broad range of *Ca* and *M*. The non-wetting fluid is injected into the rock sample already saturated with the wetting fluid. The simulations are run until the non-wetting fluid reaches the outlet boundary and breakthrough occurs. The saturation of non-wetting fluid at breakthrough time for these cases are reported in Table 1.

**Table 1:** Various simulation cases with different viscosity ratios *M* and capillary numbers *Ca*.

Case	log *M*	log *Ca*	S_nw
1	−1.17	−1.69	0.144
2	−1	−0.10	0.151
3	−0.7	−0.87	0.178
4	0	−0.48	0.234
5	−0.3	−2.19	0.242
6	0	−2.03	0.268
7	1	−2.24	0.426
8	1.18	−1.81	0.467
9	1.18	−2.14	0.501
10	1.3	0.45	0.493
11	2	0.44	0.593
12	2	−0.56	0.596

**Figure 1:**
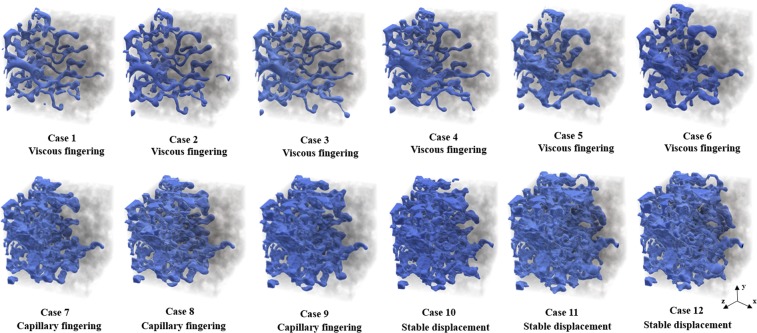
Non-wetting fluid distribution in the entire rock sample at breakthrough time for three different displacement regimes including viscous fingering (cases 1–6), capillary fingering (cases 7–9) and stable displacement (cases 10–12). The invading non-wetting fluid and the solid skeleton are shown in blue and gray, respectively.

According to the fluid patterns obtained from various simulation cases, three types of displacement patterns including capillary fingering, viscous fingering and stable displacement are identified. Figure 1 represents the non-wetting fluid distributions in the entire rock sample for twelve different cases (see Table 1) at their breakthrough times. Different displacement patterns are imposed by various capillary numbers and viscosity ratios. Cases 1 to 6 are representative of the patterns of non-wetting fluid distribution in the regime of viscous fingering. Viscous fingering illustrates an unstable displacement pattern and mostly occurs at low viscosity ratios and high capillary numbers. The fingers tend to follow preferential flow paths. As the capillary number decreases or the viscosity ratio increases, the movement patterns shifts to the capillary fingering regime (see cases 7 to 9). In this regime, the fingers slowly propagates in different directions. Thus, the breakthrough time and the final saturation of non-wetting fluid are higher than those ones for viscous fingering regime. The last three cases display stable displacement patterns in the non-wetting fluid distribution. These cases show high saturation of the non-wetting fluid at breakthrough time and basically occur at large viscosity ratios and capillary numbers. Thus, this type of displacement provides the highest drainage efficiency.

**Figure 2:**
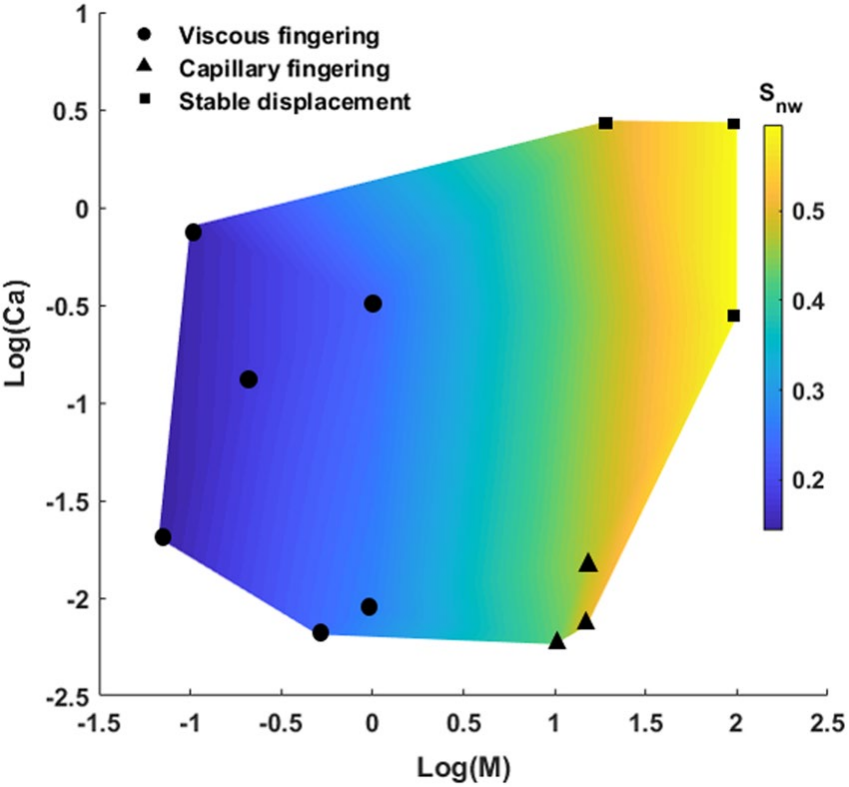
Phase diagram representing the displacement patterns and non-wetting phase saturation profile at breakthrough time as a function of viscosity ratio (*M*) and capillary number (*Ca*). The black dots indicates the simulation results.

Figure 2 depicts the displacement pattern distributions in the log *Ca*-log *M* phase diagram. The phase diagram is qualitatively consistent with those obtained experimentally by Lenormand *et al*.^19^ and Zhang *et al*.^22^ The locations of the displacement regimes is almost the same as those illustrated before for two-dimensional micromodels^19,22^. However, the capillary numbers and viscosity ratios, which define different displacement regimes in our phase diagram are different from those obtained by previous studies. It is worth noting that the classic phase diagram presented by Lenormand *et al*.^19^ only provides a qualitative presentation of the displacement patterns. Capillary number (*Ca*) and viscosity ratio (*M*) are not the only parameters that define the boundaries of displacement patterns. The heterogeneity of the pore structure, pore size distribution, porosity, pore connectivity as well as the scaling effect (size of the sample) play significant roles in boundaries of the stability zones and crossover regions in the phase diagram. Although our phase diagram could capture three distinct displacement patterns for a heterogeneous sample, the boundaries between the displacement zones differ from the classic phase diagram that has been generated for homogeneous samples through previous studies^19,22^ (see Fig. S4). Variability in pore size distribution arising from the heterogeneous nature of the pore geometry could cause the coexistence of different displacement mechanisms and, hence, limit the size of the regions of each flow regime in the phase diagram.

Additionally, wettability is another factor that could have a strong impact on the governing capillary force and stability of the displacement interface^20,52^. There have been some attempts to extend the classic phase diagram under different wettability conditions. For instance, Hu *et al*.^53^ investigated the transition of displacement fronts from fingering to stable flow influenced by wettability. Their phase diagram illustrates the crossover from corner flow to cooperative filling (stable displacement) mechanism under strong imbibition conditions (*θ* < 45 ). Thus, further investigation on characterization of flow regimes in natural rock samples is required to gain an insight into how the type and order of heterogeneity and wettability of rock formation control the displacement patterns and their mapping on phase diagram. Besides the mentioned factors, dimentionality of the system could be another reason for the discrepancy between our results and previous studies^19,22^. The real pore structure and its connectivity can be accurately captured in a three-dimensional realization of the porous sample. Therefore, it is expected that our phase diagram to be different from those obtained for a two-dimensional homogeneous sample.

To better clarify the effect of heterogeneity and dimensionality of the system on the displacement patterns and their mapping on the phase diagram, we performed two-phase flow simulations in a two-dimensional (2D) homogeneous porous medium. The locations of the simulation results for three different displacement patterns (viscous fingering, capillary fingering and stable displacement) have been presented in log *Ca*-log*M* phase diagram shown in Supplementary Information (Fig. S4). Note that the locations of different displacement regimes are placed within the regions that are defined by previous studies^19,22^ for a homogeneous medium.

It is also worth mentioning that it is not possible to define sharp boundaries in the phase diagram due to the existence of crossover zones among the displacement patterns^54^. In the crossover zones, two displacement patterns might occur simultaneously. The saturation of non-wetting fluid at breakthrough time for various displacement regimes has been mapped on the phase diagram (Fig. 2). The highest saturation and hence, the most efficient displacement corresponds to the stable displacement regime.

To quantify the two-phase displacement characteristics, the effect of capillary number and viscosity ratio on flow patterns will be discussed further in the next Sections.

### Effect of viscosity ratio

**Figure 3:**
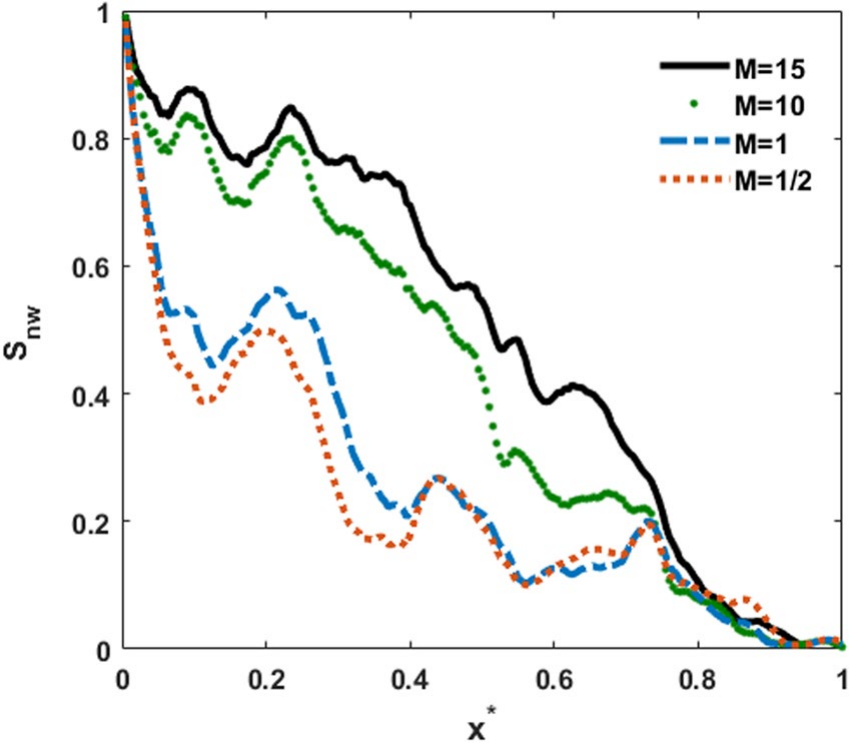
Saturation profile of non-wetting phase along the flow direction (x direction) at breakthrough time for various viscosity ratios. x^*^ is the normalized front distance from the inlet (x^*^ = *x*/*L*, where L is the length of the sample in the x direction).

We study the effect of viscosity ratio (*M*) on the dynamics of two-phase displacement in a 3D sample of Tuscaloosa sandstone. For this purpose, we focus on cases 5, 6, 7 and 9, which represents viscosity ratios of *M* = 1/2, 1, 10 and 15, respectively. In these cases, the interfacial tension, contact angle and viscosity of the wetting phase are selected as 0.001, 0° and 0.01 (in lattice units). The viscosity of the non-wetting phase is defined based on the viscosity ratio. The simulations are run until the breakthrough occurs and the invading fluid (i.e., non-wetting phase) front reaches the outlet boundary. Once breakthrough happens, the saturation of non-wetting phase is extracted. Figure 3 presents the saturation profile of the non-wetting invading fluid (*S*_*nw*_) along the flow direction (i.e., x direction) for different viscosity ratios at the breakthrough time. x^*^ is the normalized front distance from the inlet (x^*^ = *x*/*L*, where L is the length of the sample in the x direction). The results show that at locations near the inlet, as well as the outlet, the saturation distribution is almost the same for all viscosity ratios. Generally, the saturation of the non-wetting invading fluid increases as the viscosity ratio increases.

**Figure 4:**
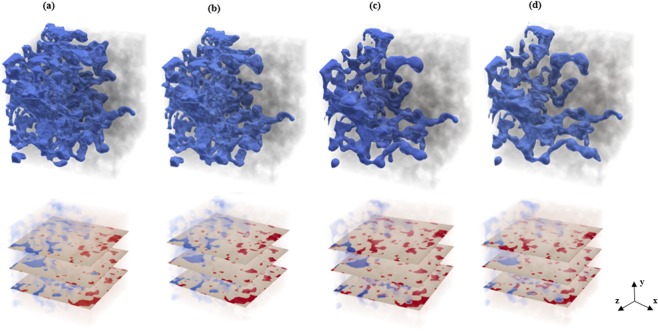
Non-wetting fluid distribution at breakthrough time in the entire sample (top) and three different slices along the flow direction (bottom) at various viscosity ratios of (**a**) *M* = 15, (**b**) *M* = 10, (**c**) *M* = 1 and (**d**) *M* = 1/2. The invading non-wetting and defending wetting fluids and the solid skeleton are shown in blue, red and gray, respectively.

Figure 4 depicts the distribution of the non-wetting fluid at the breakthrough time in the entire sample and three different slices along the flow direction for various viscosity ratios as the non-wetting fluid is injected from the left side of the sample. These phase distributions represent the presence of fingering in every case. Different displacement patterns for the invading fluid are basically associated with various displacement mechanisms and arise from various capillary numbers. As the viscosity ratio increases, the fingers of the invading fluid occupy a larger portion of the pore bodies that they pass through, as can be seen in Fig. 4. For the lowest viscosity ratio, most of the invading fingers are poorly connected and mainly move forward toward the outlet boundary. This type of displacement pattern is indicative of the viscous fingering in which the viscosity force is dominant^22^.

**Figure 5:**
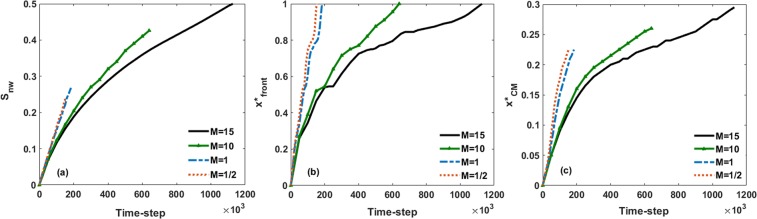
Time evolution of non-wetting fluid (**a**) saturation, (**b**) normalized front location and (**c**) normalized length of center of mass for various viscosity ratios including *M* = 15, *M* = 10, *M* = 1 and *M* = 1/2. Note that the time marching is constant throughout the entire simulations.

Figure 5(a) depicts the time variation of the non-wetting fluid saturation until breakthrough occurs. As the results indicate, the total non-wetting saturation at breakthrough is 0.501, 0.426, 0.268 and 0.242 for *M* = 15, 10, 1 and 1/2, respectively. As the viscosity ratio decreases, the breakthrough time is shorter.

The time variation of normalized frontal position of non-wetting fluid that indicates the most advanced tip of the interface is shown in Fig. 5(b). The gradient of the front position demonstrates the speed of the invading fluid advancement. It is clear that the front advances faster at a lower viscosity ratio. In other words, at lower viscosity ratios, the front position of the non-wetting phase reaches the deep parts of the medium faster, leading to an earlier breakthrough and a lower displacement efficiency. Higher velocity causes a rapid increase in the non-wetting fluid saturation when the front moves toward the outlet as is clear in Fig. 5(a). For the case with the highest viscosity ratio (*M* = 15), the slope of the front position in some time intervals in the middle part of the plot is smaller than the slope for other time steps. While the saturation of the non-wetting fluid increases monotonically (Fig. 5(a)). This behavior is attributed to the transverse and even Haines jump backward movement in the displacement regime^55,56^. This type of displacement pattern arises from the fluctuations in the pressure gradient transverse to the flow direction^57^. By comparing the displacement patterns at two different time steps, as shown in Fig. S5, the lateral and backward movement of the non-wetting fluid front can be detected. This type of movement implies that capillary fingering is the dominant displacement regime^56^.

We also calculated the length of the center of mass (${x}_{CM}^{\ast }$) of the invading pores, which shows the mean invasion length in the sample. The time evolution of ${x}_{CM}^{\ast }$ from injection to breakthrough for various viscosity ratios is shown in Fig. 5(c). The results suggest that at the breakthrough time, the center of mass is located at ${x}_{CM}^{\ast }$ = 0.29, 0.26, 0.23 and 0.23 for *M* = 15, 10, 1 and 1/2, respectively. Thus, at breakthrough for every case the non-wetting fluid is accumulated mostly at depths closer to the inlet. The spatial profiles of the non-wetting phase saturation along the sample length (Fig. 3) and the saturation distributions in Fig. 4 also validate this finding. For instance, it is clear that even though the final saturation of the invading fluid for the largest viscosity ratio is almost two times that of the final saturation for the smallest viscosity ratio, their center of mass locations do not vary significantly. This is due mainly to the existence of transverse and backward movements for the largest viscosity ratio. This type of displacement mechanism causes a few fingers to form in the regions close to the outlet. Hence, even for a high viscosity ratio, the center of mass is accumulated mostly in the locations closer to the inlet than to the outlet. In general, the results above indicate that the saturation and dynamic advancement of the invading fluid are highly influenced by the viscosity ratio.

### Effect of capillary number

**Figure 6:**
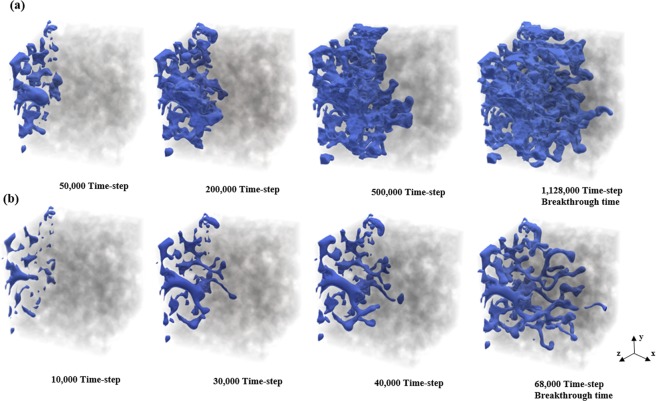
Time evolution of non-wetting phase distribution along the flow direction (x direction) for various capillary numbers (**a**) *Ca* = 7.2e-3 and (**b**) *Ca* = 8e-1. The invading non-wetting fluid and the solid skeleton are shown in blue and gray, respectively.

To better understand the effect of *Ca* on the displacement patterns, we focus on cases 2 and 9, and discuss the effect of the capillary number on the two-phase displacement and non-wetting phase saturation in detail. The simulations are run until the non-wetting phase reaches the outlet boundary and breakthrough occurs. The time evolution of the distribution of the non-wetting fluid from injection to breakthrough in the entire sample for various capillary numbers (*Ca* = 7.2e-3 and *Ca* = 8e-1) is shown in Fig. 6. Fingering can be clearly observed in the invading phase distribution, which is attributed mainly to the heterogeneous nature of the porous structure. According to these displacement patterns, for high *Ca*, due to the dominant viscous forces the fingers have thinner shapes and follow preferential paths. Thus, the case with *Ca* = 8e-1 is indicative of a viscous fingering displacement regime. The dominant viscous force causes the breakage of some fingers and, hence, the formation of some trapped blobs of the non-wetting phase in the medium^10,58,59^, which can be seen in Fig. 6(b). However, at low *Ca*, the fingers tend to grow in various directions, and the movement of the invading fluid from the inlet to the outlet is more stable. Due to the dominant capillary force at low *Ca*, the invading fluid mostly progresses through the larger pore throat; hence, the thicker fingers grow in the medium. Sometimes fingers invade backward as time progresses. For slow displacements (low *Ca*), the capillary threshold distribution inside the medium overcomes the viscous pressure gradient and the displacement patterns are controlled by the fluctuations of the capillary threshold^60,61^.

**Figure 7:**
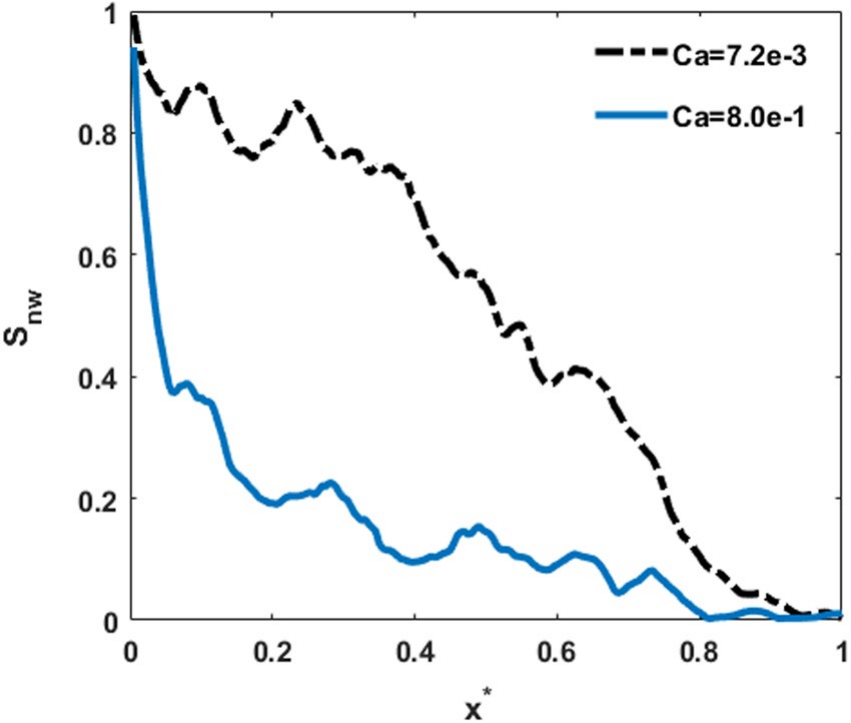
Saturation profile of non-wetting phase along the flow direction (x direction) at breakthrough time for various capillary numbers *Ca* = 7.2e-3 and *Ca* = 8e-1.

Figure 7 represents the spatial distribution of the invading fluid saturation along the sample length at the breakthrough time for different capillary numbers. As the capillary number increases, the saturation of the invading fluid is lower at a specific location from the inlet. As the results demonstrate, there is a sharp decrease in the saturation profile for the case with high capillary number, *Ca* = 8e-1. This behavior indicates that the displacement occurs in the form of viscous fingering. The phase distribution pattern shown in Fig. 6 exhibits this phenomenon. As mentioned earlier, during the drainage with a high capillary number, the snap-off of the non-wetting fluid from the main invading front leads to the formation of isolated droplets in the medium. We calculated logarithm of the non-wetting fluid saturation profile along the sample length at three different time steps (result has not been shown). The results indicate that there are some sharp variations in the saturation profiles at high depths. These jumps are attributed to the existence of isolated droplets and happens at depths where the isolated droplets have been observed.

**Figure 8:**
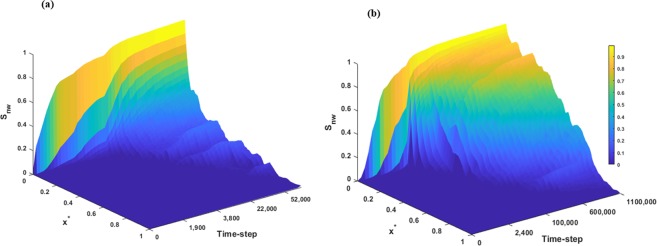
Time evolution of the non-wetting phase saturation at different planes along the flow direction for various capillary numbers (**a**) *Ca* = 8e-1 and (**b**) *Ca* = 7.2e-3.

We also calculated the time variation of the non-wetting phase saturation in the entire sample for the two capillary numbers, *Ca* = 7.2e-3 and 8e-1. Figure 8 shows the saturation profile of the invading fluid at different planes along the flow direction at various time steps. The results indicate an earlier breakthrough for the high-*Ca* case compared to the low-*Ca* case. It is obvious that for both cases, the saturation of non-wetting phase increases as time proceeds. Even though the trend of non-wetting saturation profile for the first few planes are similar for both *Ca* cases, the higher saturation in the middle planes for low *Ca* case is clear in Fig. 8. In other words, the case with the higher *Ca* yields the lower saturation of invading fluid in the greater part of the sample.

### Fractal dimension of invasion patterns during two-phase displacement

Previous studies have shown that an unstable invasion pattern during two-phase displacement in porous media is fractal^60,62,63^. The fractal dimension is representative of the geometrical complexity of the displacement patterns and demonstrates how the invading clusters occupy the pore space. The front shape is highly dependent on the capillary number, viscosity ratio and the heterogeneity of the medium^22^. The fractal dimension provides information regarding the characteristics of the developed front and determines its complexity. In the traditional modeling of multiphase flow in porous media, it is assumed that the saturation front advances linearly with time. However, it is found that at small-scale flow, the saturation front displaces non-linearly with time. This non-linearity arises from the fingering phenomena and the fractal nature of their patterns at pore-scale^57^. Thus, we investigated the fractal nature of the non-wetting phase saturation profile during immiscible displacement in the medium. We have calculated the fractal dimension of the invaded cluster of the non-wetting fluid for various capillary numbers and viscosity ratios to better characterize the morphology of the invasion patterns at various conditions. While previous studies have calculated the fractal dimension of two-dimensional, two-phase flow displacement patterns, the estimation of the fractal dimension of 3D clusters of displacement structures in porous media is rare and there is a need for further study. Thus, calculating the evolution of the fractal dimension of 3D displacement patterns in this study provides a realistic insight into quantifying the front shape and its degree of disorder during the drainage process.

Using the non-wetting phase saturation patterns during two-phase immiscible displacement, we have estimated the fractal dimension for 3D non-wetting fluid clusters. The fractal dimension is calculated using the standard box-counting method^64^. In this method, the displacement structures are covered with boxes of size *r*, and the number of boxes, *N*(*r*) that covers the structure is counted. The number of boxes *N*(*r*) as a function of box size *r* follows a power law:

1 $$N(r)\propto {r}^{-{D}_{f}}$$

The number of boxes *N*(*r*) of size *r* is plotted as a function of the box size *r* in a log-log plot and the slope of the curve represents the fractal dimension *D*_*f*_^64,65^. For instance, Fig. S9 shows the logarithm of counted boxes versus logarithm of box size for a non-wetting fluid configuration taken from the simulation of case 9. The fractal dimension *D*_*f*_ is calculated using the slope of the curve.

As is clear from the non-wetting phase saturation profiles (Figs 4 and 6), the fingering makes the front shape more complex than a piston-like interface. We have calculated the fractal dimension of the 3D invading fluid clusters at breakthrough time. The front fractal dimension for the case with a lower capillary number (*D*_*f*_ = 2.54 ± 0.14) is higher than the other case with higher capillary numbers (*D*_*f*_ = 2.33 ± 0.11). This is due mainly to the more trapping, transverse and Haines jump backward movement, which causes more complex patterns and a larger fractal dimension in the capillary fingering regime (see Fig. 6(a)).

**Figure 9:**
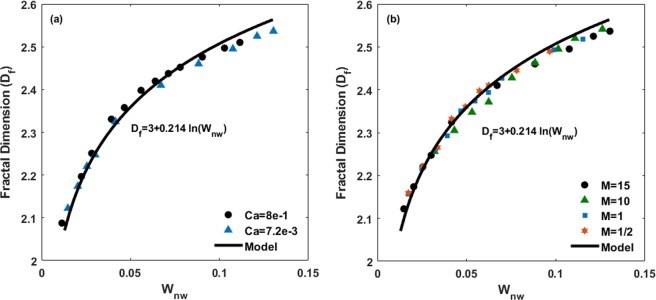
Non-wetting phase content (*W*_*nw*_) dependence of fractal dimension *D*_*f*_ for various (**a**) capillary numbers and (**b**) viscosity ratios.

The relationship between fractal dimension *D*_*f*_ with the non-wetting fluid saturation *S*_*nw*_ or fluid content *W*_*nw*_ (*S*_*nw*_ × *ϕ*, where *ϕ* is the porosity) in a porous medium can be described by^61^

2 $${D}_{f}=d+m\,\mathrm{ln}({S}_{nw}\varphi )=d+m\,\mathrm{ln}({W}_{nw})$$

where d represents the Euclidean dimension and m is a parameter that is dependent on the pore-size distribution of the medium. The logarithmic dependence of the fractal dimension *D*_*f*_ of the displacement patterns on the non-wetting fluid content for various capillary numbers and viscosity ratios is shown in Fig. 9. Remarkably, the data represents an excellent agreement with Eq. (2). According to Fig. 9, this agreement is not affected by the viscosity ratio and capillary number, and all data collapse on the same curve. Thus, it implies that the fractal dimension *D*_*f*_ is a universal function of the non-wetting phase content.

## Summary

In this study, micro-CT images of Tuscaloosa sandstone were applied to simulate displacement during drainage using the lattice Boltzmann method. The major achievement of our study was to mimic two-phase flow displacement in a realistic three-dimensional rock model, which enabled us to gain a better insight into real reservoir problems. A series of two-phase displacement simulations was performed and three typical flow patterns are identified and located on the *Ca*-*M* phase diagram. We also investigate the effect of viscosity ratio (*M*) and capillary number (*Ca*) on the displacement pattern and phase distribution in the natural rock sample. The evolution of saturation, the location of the displacement front and the length of the center of mass of the invading fluid have been studied for cases having various viscosity ratios and capillary numbers. A low viscosity ratio leads to the lower saturation of the non-wetting fluid at breakthrough time, non-compact displacement patterns, the tearing off of the finger heads and the formation of isolated droplets. A high viscosity ratio causes the slow movement of the displacement front, a higher non-wetting fluid saturation at breakthrough and displacement efficiency. It is also found that the length of the center of mass of the invading fluid is not a strong function of the displacement regime. In order to better quantify the geometry of the displacement patterns during each test, we have computed the fractal dimension of the invading phase cluster. The three-dimensional fractal dimension of the displacement patterns at breakthrough time was found to be larger for the test with lower capillary number and higher viscosity ratio, which is due mainly to the more complex displacement patterns arising from transverse and Haines jump backward movement. We also found that the fractal dimension of the displacement clusters is a universal function of the non-wetting fluid saturation. Finally, the present multiphase LB model is a promising scheme for the application of CO_2_ sequestration and can be used for the evaluation of CO_2_ sweep efficiency and the storage capacity of a reservoir at various displacement regimes.

## Methods

### Multiphase lattice Boltzmann method

In this study, we adopt an optimized Colour-Gradient LB model^44^ for the simulation of a two-phase flow in a rock sample. A D3Q19 multiple-relaxation-time (MRT) scheme has been applied. In the LB model, the fluid particle distribution function, *f*_*i*_(*x*, *t*), undergoes the propagation and collision operators, which are described by the following equation^66,67^

3 $${f}_{i}({\rm{x}}+{{\rm{e}}}_{i}{\rm{\Delta }}t,t+{\rm{\Delta }}t)-{f}_{i}({\rm{x}},t)={{\rm{\Omega }}}_{i},i=\mathrm{0,}\ldots ,\,\mathrm{18,}$$

where Δ*t* represents the time step in lattice units, Ω_*i*_ is the collision operator and e_*i*_ are the velocity basis vectors. The velocity set *e*_*i*_ is given as

4 $${e}_{i}=\{\begin{array}{ll}\mathrm{(0,}\,\mathrm{0,}\,\mathrm{0),} & i=\mathrm{0,}\\ c(\pm \,\mathrm{1,}\,\mathrm{0,}\,\mathrm{0)},\,c\mathrm{(0,}\,\pm \mathrm{1,}\,\mathrm{0),}\,c\mathrm{(0,}\,\mathrm{0,}\,\pm \,\mathrm{1),} & i=\mathrm{1,}\,\mathrm{2,}\ldots ,\,\mathrm{6,}\\ c(\pm \,\mathrm{1,}\,\pm \,\mathrm{1,}\,\mathrm{0),}\,c(\pm \,\mathrm{1,}\,\mathrm{0,}\pm \,\mathrm{1),}\,c\mathrm{(0,}\,\pm \,\mathrm{1,}\,\pm \,\mathrm{1),} & i=\mathrm{7,}\,\mathrm{8,}\ldots ,\,18.\end{array}$$

$c=\frac{{\rm{\Delta }}x}{{\rm{\Delta }}t}$ is the lattice velocity and Δ*x* is the grid spacing.

The effect of interfacial tension *σ* incorporates into the collision operator, which has been described by a multiple-relation-time (MRT) scheme as follows:

5 $${\rm{\Omega }}={{\rm{M}}}^{-1}{\rm{S}}[({\rm{M}}f)-{{\rm{m}}}^{eq}],$$

where M is the transformation matrix, which transforms the distribution function *f* to the moment space m as shown below:

6 $${\rm{m}}={\rm{M}}f,\,{{\rm{m}}}^{eq}={\rm{M}}{f}^{eq},$$

where *f*^ *eq*^ and m^eq^ are equilibrium functions at distribution and moment space, respectively.

The density *ρ*, energy *e*, energy-square *ε*, momentum components, *j*_*x*_ = *ρ*_0_*u*_*x*_, *j*_*y*_ = *ρ*_0_*u*_*y*_, and *j*_*z*_ = *ρ*_0_*u*_*z*_ (*ρ*_0_ is a constant reference density), heat flux flux components, *q*_*x*_, *q*_*y*_ and *q*_*z*_, and stress tensor components *p*_*xx*_, *p*_*ww*_ = *p*_*yy*_ − *p*_*zz*_, *p*_*xy*_, *p*_*yz*_, *p*_*xz*_ are used in moment vector m, as shown below:

7 $${\rm{m}}=(\rho ,e,\varepsilon ,{j}_{x},{q}_{x},{j}_{y},{q}_{y},{j}_{z},{q}_{z},{p}_{xx},{\pi }_{xx},{p}_{ww},{\pi }_{ww},{p}_{xy},{p}_{yz},{p}_{xz},{m}_{x},{m}_{y},{m}_{z}),$$

The detail of moment m and transformation matrix M can be found in previous studies^45,68^. Th equilibrium moment vector m^eq^ is calculated by taking the effect of interfacial tension into consideration, as shown below^25,45^

8a $${m}_{0}^{eq}=\rho ,$$

8b $${m}_{1}^{eq}={e}^{eq}=-\sigma |C|,$$

8c $${m}_{3}^{eq}={j}_{x},$$

8d $${m}_{5}^{eq}={j}_{y},$$

8e $${m}_{7}^{eq}={j}_{z},$$

8f $${m}_{9}^{eq}=3{p}_{xx}^{eq}=\frac{1}{2}\sigma |C|(2{n}_{x}^{2}-{n}_{y}^{2}-{n}_{z}^{2}),$$

8g $${m}_{11}^{eq}={p}_{zz}^{eq}=\frac{1}{2}\sigma |C|(2{n}_{y}^{2}-{n}_{z}^{2}),$$

8h $${m}_{13}^{eq}={p}_{xy}^{eq}=\frac{1}{2}\sigma |C|({n}_{x}{n}_{y}),$$

8i $${m}_{14}^{eq}={p}_{yz}^{eq}=\frac{1}{2}\sigma |C|({n}_{y}{n}_{z}),$$

8j $${m}_{15}^{eq}={p}_{xz}^{eq}=\frac{1}{2}\sigma |C|({n}_{x}{n}_{z}),$$

8k $${m}_{2}^{eq}={m}_{4}^{eq}={m}_{6}^{eq}={m}_{8}^{eq}={m}_{16}^{eq}={m}_{17}^{eq}={m}_{18}^{eq}=\mathrm{0,}$$

The definition of *C*, *n*_*x*_, *n*_*y*_ and *n*_*z*_ are presented in equations 15 and 17. The collision matrix *S* is a diagonal matrix

9 $${\rm{S}}={\rm{diag}}({s}_{0},\ldots ,{s}_{18}),$$

The matrix S has 19 relaxation parameters *S*_*i*,*i*_, also known as the eigenvalues of the collision matrix M^−1^SM as follows:

10 $$S=({s}_{e},\,{s}_{\xi },\,\mathrm{0,}\,{s}_{q},\,\mathrm{0,}\,{s}_{q},\,\mathrm{0,}\,{s}_{q},\,{s}_{\nu },\,{s}_{\pi },\,{s}_{\nu },\,{s}_{\pi },\,{s}_{\nu },\,{s}_{\nu },\,{s}_{\nu },\,{s}_{m},\,{s}_{m},\,{s}_{m},\,\mathrm{0)},$$

The relaxation parameter *s*_*ν*_ is defined as:

11 $${s}_{\nu }=\frac{2}{6\frac{\nu }{{c}^{2}{\rm{\Delta }}t}+1},$$

where *ν* is the kinematic viscosity. The remaining parameters of the matrix S must lie between 0 and 2 to improve the stability. The optimal values of these parameters can be found in previous studies^69,70^.

The macroscopic properties, including density (*ρ*) and velocity (*u*), are calculated using the following relations:

12 $$\rho =\sum _{i}\,{f}_{i},$$

13 $$\rho u=\sum _{i}{e}_{i}{\rho }_{i},$$

An optimized colour-gradient approach^44,45^ was applied to model the two-phase flow. This model provides stable solutions for a low capillary number and a high viscosity ratio. An order parameter *ϕ* is defined as follows:

14 $$\varphi =\frac{{\rho }_{w}-{\rho }_{nw}}{{\rho }_{w}+{\rho }_{nw}},$$

where *ρ*_*w*_ and *ρ*_*nw*_ are the dimensionless density field of the wetting and non-wetting phase, respectively. The order parameter *ϕ* represents the fluid phase distribution and its value is −1 and +1 for the non-wetting and wetting phase, respectively. Its value varies between −1 and 1 at the diffusive interface of wetting and non-wetting phase. The colour gradient of the phase field is calculated as:

15 $$C(t,x)=\frac{3}{{c}^{2}{\rm{\Delta }}t}\sum _{n=1}{w}_{i}{e}_{i}\varphi (t,x+{e}_{i}{\rm{\Delta }}t),$$

where *w*_*i*_ are the weight coefficients as follows:

16 $$\{\begin{array}{ll}\frac{1}{3}, & i=\mathrm{0,}\\ \frac{1}{18}, & i=1,2,\ldots ,6,\\ \frac{1}{36}, & i=7,8,\ldots ,18.\end{array}$$

The normalized gradient, which represents the orientation of the interface between the phases, is defined as:

17 $${n}_{k}=\frac{{C}_{k}}{|C|},$$

where *k* represents either the wetting phase or the non-wetting phase.

The LB model has been applied to compute the advection of density fields *ψ* = (*ρ*_*w*_, *ρ*_*nw*_) as follows:

18 $${g}_{i}({\rm{x}}+{{\rm{e}}}_{i}{\rm{\Delta }}t,t+{\rm{\Delta }}t)={g}_{i}^{eq}(\psi (t,x),u(t,x)),i=\mathrm{0,}\ldots ,\,\mathrm{18,}$$

${g}_{i}^{eq}$ is the equilibrium distribution function and given by

19 $${g}_{i}^{eq}(\psi ,u)={w}_{i}\psi (1+\frac{3}{{c}^{2}}{e}_{i}\mathrm{.}u),i=\mathrm{0,}\ldots ,\,\mathrm{18,}$$

The velocity profile *u* is calculated by the MRT LBM, which is described above. Then, the densities of the wetting and non-wetting fluids are calculated by $\psi ={\sum }_{i}{g}_{i}$. Finally, a recoloring scheme is applied to redistribute the distribution function *g*_*i*_ in order to minimize the diffusion near the interface and attain the separation of the two fluids. The details of the recoloring approach are described in previous studies^45,71^.

The accuracy of our LB model is validated through a series of simulations for static contact angle evaluation and capillary filling dynamics. The corresponding results are presented in Supplementary Information.

## Supplementary information

Case12

Case7

Case6

Supplementary Information
