# Supplementary material for: Pore-scale characteristics of multiphase flow in heterogeneous porous media using the lattice Boltzmann method
Source: Sci Rep. 2019 Mar 4;9:3377. doi: 10.1038/s41598-019-39741-x (PMC6399269; doi:10.1038/s41598-019-39741-x)
Supplement: Supplementary file 4 — Supplementary Information [file 41598_2019_39741_MOESM4_ESM.docx]

**Pore-scale characteristics of multiphase flow in heterogeneous porous media using the lattice Boltzmann method**

Sahar Bakhshian^1*^, Seyyed A. Hosseini^1^, Nima Shokri^2^

^1^Bureau of Economic Geology, Jackson School of Geosciences, The University of Texas at Austin, TX, 78758, USA

^2^School of Chemical Engineering and Analytical Science, The University of Manchester, Manchester, UK

Correspondence and requests for materials should be addressed to Sahar Bakhshian (email: sahar.bakhshian@beg.utexas.edu)

**Supplementary information**

The three-dimensional micro-CT images of Tuscaloosa sandstone and the digitized image stack used in the LB simulation are shown in figure S1.


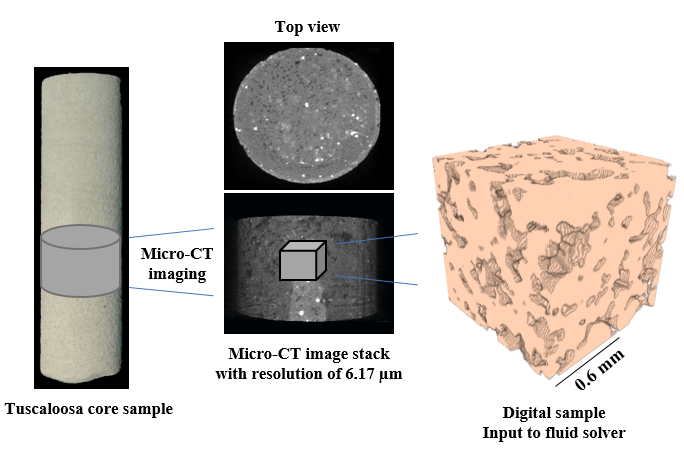


**Figure S1.** Original Tuscaloosa core sample, its micro-CT image stack and the binary 3D image as the input to the LB model.

**Analysis of sample heterogeneity**

The pore size distribution of the digital rock used in the simulations is presented in figure S2.


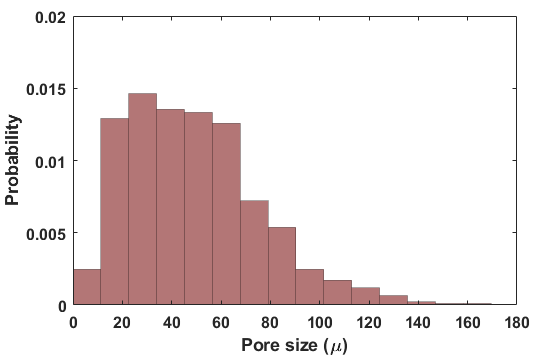


**Figure S2**. Pore-size distribution of the Tuscaloosa sandstone used in the present study.


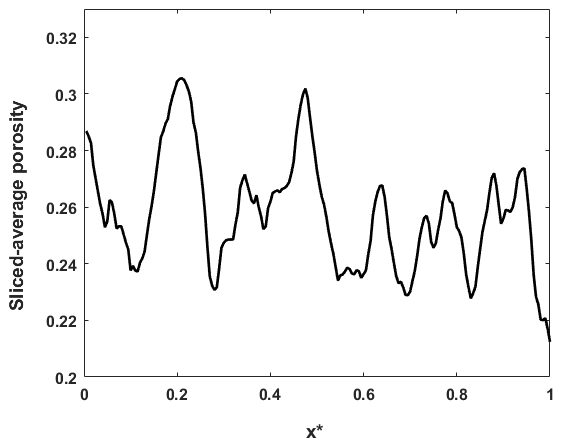


**Figure S3**. Sliced-average porosity profile along the sample length (x direction). x* is the normalized front distance from the inlet (x*=x/L, where L is the length of the sample in the x direction).

The pore size of the sample is statistically analyzed and its average, standard deviation, skewness and kurtosis have been reported in Table S1.

**Table S1**. Analysis of sample heterogeneity.

| Pore-size average (µ) | Pore-size standard deviation (µ) | Pore-size skewness | Pore-size kurtosis |
| --- | --- | --- | --- |
| 48.94 | 26.89 | 0.85 | 3.72 |

**Displacement patterns in a 2D homogenous medium**


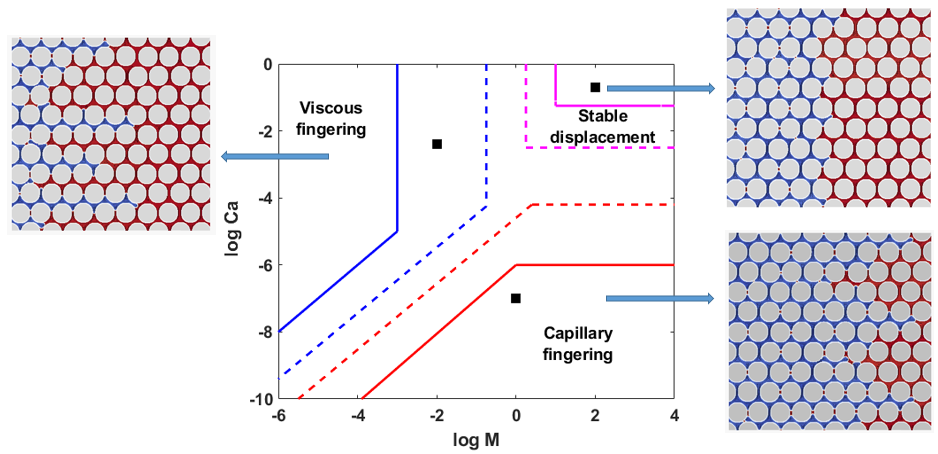


**Figure S4**. Phase diagram representing the displacement patterns in a 2D homogenous medium. Note that the square symbols correspond to the present LB simulations in a 2D homogenous sample. The solid and dashed lines represent the boundaries of stability zones obtained by Lenormand et al.^1^ and Zhang et al.^2^.

**Model Validation**

**Static contact angle evaluation.** To verify the accuracy of our two-phase flow LB model, the wetting phenomenon is simulated for a wide range of surface wettability. To adjust a contact angle between the fluid and the solid skeleton, an order parameter $\emptyset_{solid}$is set to the solid nodes that control the surface wettability. The equilibrium contact angle is defined as^3^

$\cos\left( \theta\right)= \emptyset_{solid} (1)$

where $\theta$ is the contact angle. The order parameter $\emptyset_{solid}$ varies between -1 and +1, where -1 represents a non-wetting or hydrophobic surface and +1 indicates a wetting or hydrophilic one. Assigning the order parameter to the solid nodes gives rise to a colour gradient with the fluid nodes and implements the interaction between the fluid and solid nodes.

Simulations have been performed in a $200\times200\times200$lu^3^ (lu means lattice unit) computational domain in which two immiscible fluids are placed above a solid surface with various wettability. The interfacial tension is fixed at $\sigma=0.01$ (in lattice unit). The viscosity ratio, which is assumed to be the ratio of the viscosity of the non-wetting fluid to the wetting fluid viscosity ($M={\mu_{nw}}/{\mu_{w}}$ , where $\mu_{nw}$ and $\mu_{w}$ are the dynamic viscosity of the advancing non-wetting and displaced wetting fluids, respectively), is set to 1/4. A periodic boundary condition is adopted for both the left and right boundaries, whereas the bottom and top boundaries are considered to be solid walls. In the simulations, the wetting phase is initialized with a cubic configuration that is in contact with the bottom wall. The non-wetting phase is placed in the remaining area. Through LB simulation, the wetting phase finally reaches an equilibrium state, and the final contact angle and a stable configuration form. The results of the simulations for various surface wettabilities are presented in figure S5. The parameter $\emptyset_{solid}$has been chosen as 0.86, 0.34 and -0.5 for equilibrium contact angles of 30◦, 70◦ and 120◦, respectively. It is found that higher wettability leads to a smaller contact angle. The simulated contact angles are measured via the method proposed by Huang et al.^4^ The contact angle can be analytically determined using Eq. (1). According to this equation, various contact angles are imposed by using different values of parameter $\emptyset_{solid}$. In figure S6. a, the simulated contact angles are compared with the ones calculated using Eq. (1). The results indicate that the contact angles obtained from the present LB model agree well with the theoretical values and they are totally consistent with the applied parameter $\emptyset_{solid}$. Thus, our LB model gives the accurate static contact angle


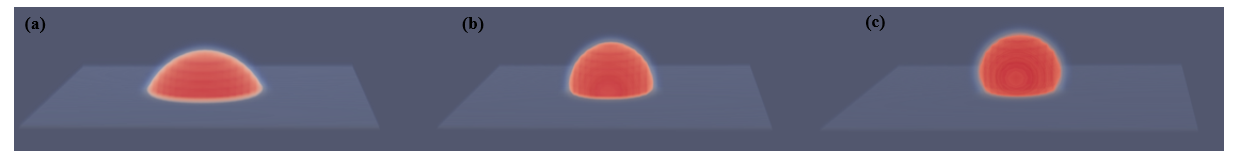


**Figure S5.** Droplet of wetting phase on a solid surface with contact angles of (a) 70^ᵒ^ (b) 100^ᵒ^ and (c) 120^ᵒ^. The red shows the wetting fluid, which is surrounded by a non-wetting fluid.


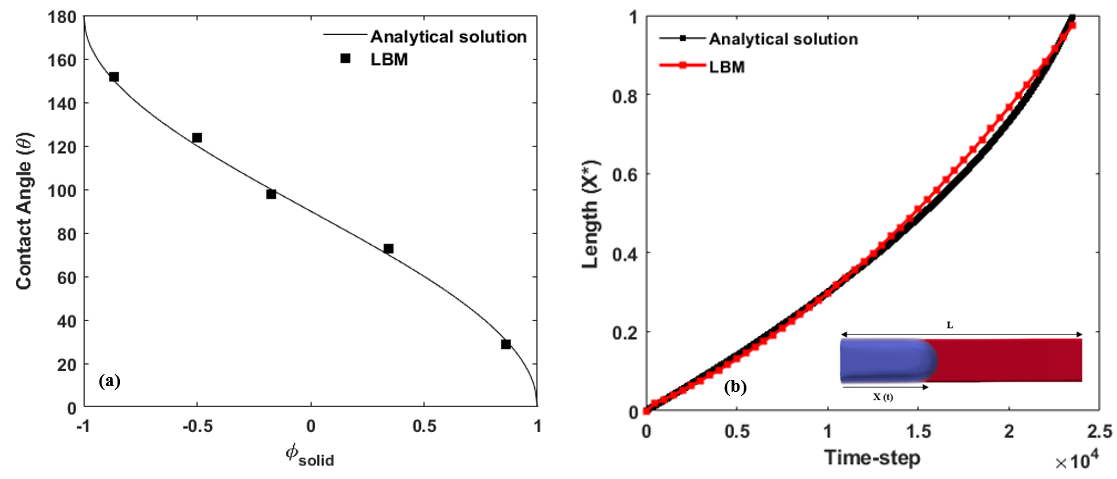


**Figure S6**. (a) Contact angles obtained from the present LB model compared with the analytical solution. (b) Evolution of the length of the non-wetting fluid invaded a capillary tube (A comparison between the result from the LB model and the one calculated using the analytical model). The Blue and red color represent the non-wetting and wetting fluid, respectively.

**Capillary filling dynamics.** To verify the LB model for capillary filling and displacement simulations, the invasion of a non-wetting fluid into a single capillary tube as well as a bundle of parallel tubes with rectangular cross sections are simulated. For the first case, we considered a three-dimensional tube of size $150\times20\times20$ lu^3^ that initially contains a wetting fluid. A schematic diagram of the simulation domain is shown in the inset of figure S6. b. Two buffer layers with a size of 10 lu have been added at the inlet and outlet of the tube. A non-wetting fluid, which is initially filled the inlet buffer layer, is driven to tube by applying a pressure gradient (∆P) between the inlet and the outlet. The analytical solution for the invaded length of the tube as a function of time t is given by^5^

$$X\left( t \right)=\frac{\mu_{w}L_{1}- \sqrt{\mu_{w}^{2}L_{1}^{2}+\frac{\left( \mu_{nw}-\mu_{w} \right)\Delta PR_{eq}^{2}}{4}}t}{\mu_{w}-\mu_{nw}} (2)$$

where $L_{1}$ is the length of the tube. $R_{eq}$is the tube radius or equivalent curvature of the tube with a rectangular cross section that is defined as

$$R_{eq}=\frac{1}{\frac{2}{L_{2}}+\frac{2}{L_{3}}} (3)$$

where $L_{2}$ and $L_{3}$ are the width and length of the rectangular cross section. In the simulation the viscosity ratio was set to 1/4, $\mu_{w}=0.12$, $\mu_{w}=0.03$. The surface tension and contact angle are considered to be 0.01 and 30^◦^, respectively. In figure S6. b, the red curve represents the simulation result for the evolution of the length of the non-wetting fluid, which invaded the capillary tube. The results obtained from the LB simulation is consistent with the analytical solution, which is calculated using Eq. (2).

In the next set of simulations, the drainage of a non-wetting fluid into a bundle of capillary tubes is studied. The computational domain is $80\times20\times55$ lu^3^, as shown in figure S7. Two buffer layers with a size of 10 lu have been added at the inlet and outlet of the tube bundle. Initially, the capillary tubes are occupied by a wetting phase, and a non-wetting phase is injected from the buffer zone to the system by applying a pressure gradient between the inlet and outlet. The simulations are run until the system reaches a steady state. The corresponding capillary pressure of each tube is defined as^6^

$$P_{c}=\frac{2\sigma cos\theta}{R_{eq}} (4)$$

The equilibrium contact angle is fixed at 30^◦^. The viscosity ratio and surface tension are set as 1/4 and 0.01, respectively. Different pressure gradients ∆P are applied to the system. Two-dimensional snapshots of the injection simulations for various pressure gradients are shown in figure S7, where the view is along the z-axis. For the lowest pressure gradient ∆P, which is smaller than the capillary pressure of the larger tube (P_cl_), the non-wetting fluid is unable to invade into either tube. When ∆P is above the capillary pressure of any tube, the non-wetting phase can enter into that tube. The simulation results reveal the competition of capillary and viscous forces in the system. Since a larger tube leads to lower capillary pressure, the non-wetting phase can be drained out faster and more easily than in smaller tubes. In other word, larger pores have lower capillary resistance and, hence, higher permeability, and the non-wetting phase breaks though the larger pores first. By applying a pressure gradient greater than the capillary pressure of the smaller tube (P_cu_), the non-wetting fluid invades both tubes (figure S7. c).


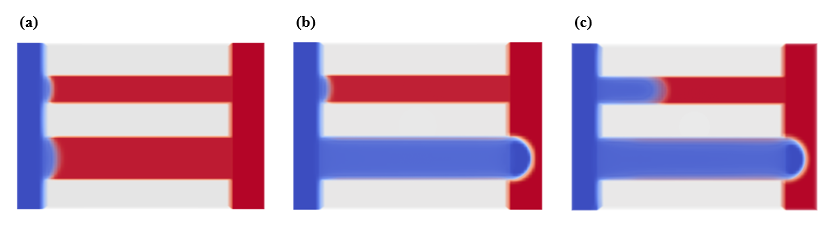


**Figure S7.** Snapshots of the injection of a non-wetting fluid into two parallel capillary tubes under various pressure gradients (a) ∆P < P_cl_, (b) P_cl_ <∆P < P_cu_ and (c) ∆P > P_cu_. P_cl_ and P_cu_ are the capillary pressures of the smaller and larger tubes, respectively. The gray, blue and red color represent the solid, non-wetting and wetting fluid, respectively.

**Backward flow in capillary fingering regime**

To represent the lateral and backward movement in the capillary fingering dominated regime, the displacement patterns of the non-wetting fluid have been shown at two different time steps (figure S8).


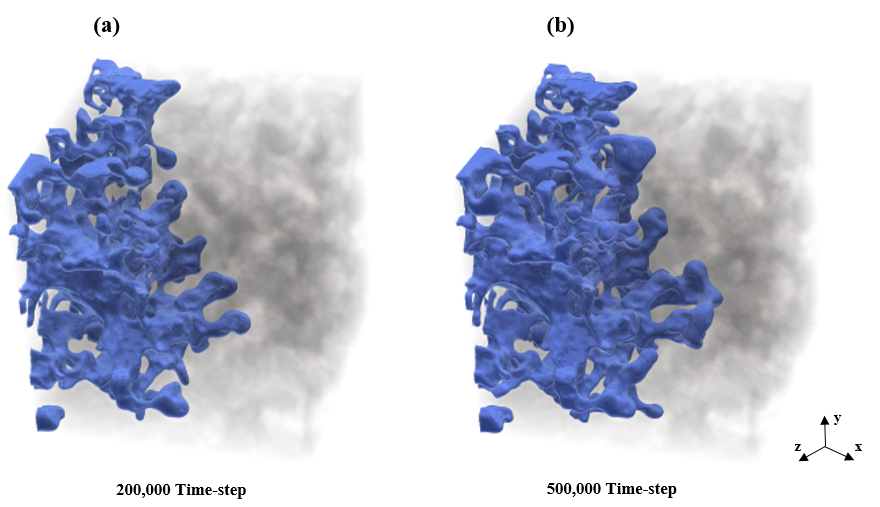


**Figure S8.** Non-wetting fluid saturation at two different time steps for M=15. The patterns are representative of transversal and backward flow.

**Fractal scaling law for 3D non-wetting fluid distribution**

The fractal dimension *D_f_* is determined by the slope of a linear fit through data on log-log plot of the box numbers N(r) versus the box size r. We have selected a non-wetting fluid configuration obtained from simulation of case 9 and demonstrated the log-log plot of the number of boxes versus the box size in figure S9.


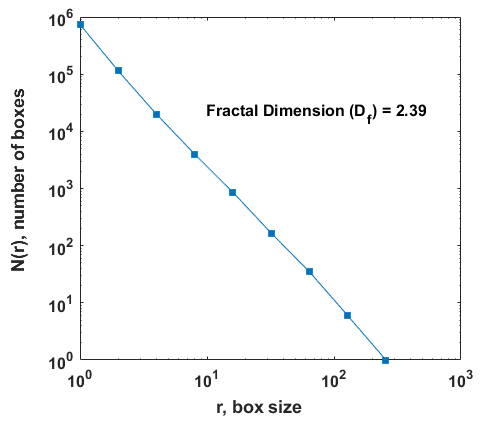


**Figure S9**. Fractal dimension of the invading (non-wetting fluid) structure taken from simulation of case 9 at a specific time. The slope of the curve represents fractal dimension, D_f_.

**References**

1. Lenormand, R., Touboul, E. & Zarcone, C. Numerical models and experiments on immiscible displacements in porous media. *J. Fluid Mech.* **189**, 165-187 (1988).
2. Zhang, C., Oostrom, M., Wietsma, T. W., Grate, J. W. & Warner, M. G. Influence of viscous and capillary forces on immiscible fluid displacement: pore-scale experimental study in a water-wet micromodel demonstrating viscous and capillary fingering. *Energy Fuels* **25**, 3493-3505 (2011).
3. Latva-kokko, M. & Rothman, D.H. Static contact angle in lattice Boltzmann models of immiscible fluids. *Phys. Rev. E* **72**, 046701 (2005).
4. Huang, H., Thorne, D.T., Schaap, M.G. & Sukop, M.C. Proposed approximation for contact angles in Shan-and-Chen-type multicomponent multiphase lattice Boltzmann models. *Phys. Rev. E* **76**, 066701 (2007).
5. Ahrenholz, B., T$\ddot{o}$lke, J., Lehmann, P., Peters, A., Kaestner, A., Krafczyk, M. & Durner, W. Prediction of capillary hysteresis in a porous material using lattice-Boltzmann methods and comparison to experimental data and a morphological pore network model. *Adv. Water Resour.* **31**, 1151-1173 (2008).
6. Dahle, H.K., Celia, M.A. & Hassanizadeh, S.M. Bundle-of-tubes model for calculating dynamic effects in the capillary pressure saturation relationship. Transp. Porous Media 58, 5-22 (2005).
